# CYP17 promoter polymorphism and breast cancer risk in males and females in relation to BRCA2 status

**DOI:** 10.1038/sj.bjc.6600839

**Published:** 2003-03-18

**Authors:** K Gudmundsdottir, S Thorlacius, J G Jonasson, B F Sigfusson, L Tryggvadottir, J E Eyfjord

**Affiliations:** 1 Molecular and Cell Biology Research Laboratory, Icelandic Cancer Society, 125-Reykjavik, Iceland; 2 Iceland Genomics Corporation, Reykjavik, Iceland; 3 Cancer Registry, Icelandic Cancer Society, 125-Reykjavik, Iceland; 4 Cancer Detection Clinic, Icelandic Cancer Society, 125-Reykjavik, Iceland; 5 Department of Pathology, University Hospital, Reykjavik, Iceland; 6 Faculty of Medicine, University of Iceland, Reykjavik, Iceland

**Keywords:** male and female breast cancer, CYP17, BRCA2

## Abstract

A T–C polymorphism in the promoter region of the CYP17 gene has been associated with male and female breast cancer risk as well as early-onset familial breast cancer. The potential role of this polymorphism was investigated in relation to breast cancer risk in Icelandic male and female carriers and noncarriers of a BRCA2 mutation. The study population consisted of 39 male and 523 female breast cancer cases and 309 male and 395 female controls. Of the cases, 15 males and 55 females carried a BRCA2 mutation. We did not find a significant association between male breast cancer risk and CYP17 genotypes. Among male breast cancer cases, the frequency of the CC genotype was higher among carriers of the 999del5 mutation (33.3%) than noncarriers (16.7%), although this difference also did not reach a statistical significance. No association was observed with breast cancer risk among females irrespective of menopausal status, stage of the disease or BRCA2 status. Our findings do not indicate a role for the CYP17 T–C polymorphism in female breast cancer, but a role in male carriers of a BRCA2 mutation could not be excluded because of the small sample size.

Breast cancer is the most common cancer among women in Iceland, with one out of every 12 women developing breast cancer in their lifetime (Tulinius *et al*, 1999). Among men, however, breast cancer is very rare, only representing about 1% of all breast cancer cases in Iceland and 0.25% of all malignant tumours in males (Jonasson *et al*, 1996).

In Iceland, only one mutation has been found in each of the BRCA genes, a rare BRCA1 mutation, D1692N (<1% of breast cancer cases) (Bergthorsson *et al*, 1998) and a more common BRCA2 mutation. The BRCA2 mutation is a 5 bp deletion in exon 9, 999del5, which leads to an early protein termination (Thorlacius *et al*, 1996). Studies have shown that this mutation is found in an estimated 0.6% of the population. In breast cancer patients, the carrier frequency is around 8% for females and 40% among males (Thorlacius *et al*, 1997). The risk of developing breast cancer varies greatly between mutation carriers, which indicates the involvement of genetic and/or environmental modifying factors (Thorlacius *et al*, 1998). Possible modifiers include enzymes involved in steroid hormone biosynthesis, since steroid hormones play an important role in the development of breast cancer.

The CYP17 gene codes for the cytochrome *P*450c17*α* enzyme, which is involved in the synthesis of androgens and oestrogens. The 5′ untranslated region of the gene contains a T (A1 allele) to C (A2 allele) polymorphism, 34 bp upstream from the initiation of translation. This base pair change creates an additional Sp1-type (CCACC) promoter motif, which has been hypothesised to lead to increased transcriptional activity and enhanced steroid hormone production (Carey *et al*, 1994). However, an *in vitro* assay was not able to confirm the creation of an Sp1 binding site (Nedelcheva Kristensen *et al*, 1999). In spite of that, two studies have observed elevated levels of sex hormones in serum in women in association with the C allele (Feigelson *et al*, 1998; Haiman *et al*, 1999).

Several studies have looked at a possible association between the promoter polymorphism in the CYP17 gene and breast cancer risk in females. One study found an increased risk of advanced breast cancer in women carrying a C (A2) allele (Feigelson *et al*, 1997). They also observed that the protective effect of older age at menarche was largely limited to women carrying the TT (A1A1) genotype. Other studies have followed with mixed results (Dunning *et al*, 1998; Helzlsouer *et al*, 1998; Weston *et al*, 1998; Bergman-Jungestrom *et al*, 1999; Haiman *et al*, 1999; Huang *et al*, 1999; Nedelcheva Kristensen *et al*, 1999; Mitrunen *et al*, 2000; Spurdle *et al*, 2000). The overall findings in these studies suggest that the CYP17 polymorphism does not have an effect on breast cancer risk in general, but may modify risk in certain subgroups. To our knowledge, only one report has stratified risk according to the BRCA status of the individual. Spurdle *et al* (2000) reported an increased risk of early-onset familial breast cancer in association with the CC genotype. This increase in risk did not seem to apply to carriers of mutations in either BRCA1 or BRCA2.

One study has been published on the possible role of CYP17 in the development of male breast cancer (Young *et al*, 1999). They compared genotype frequencies in 64 breast cancer cases to 81 controls and found an increased risk of breast cancer in association with the C (A2) allele (OR=2.10; 95% CI, 1.04–4.27). To our knowledge, there have been no published studies on a potential association between the CYP17 polymorphism and male breast cancer in BRCA2 carriers.

The current study aimed to investigate the association between the CYP17 polymorphism and breast cancer risk in Icelandic males and females. A possible association with the BRCA2 status of males and females and the stage of the disease in females was also investigated.

## MATERIALS AND METHODS

### Study population

All male breast cancer cases diagnosed in Iceland from 1955 to 2000 were included in the study, 39 in total. The 29 cases diagnosed in the time period from 1955–1994 have all been previously described (Jonasson *et al*, 1996). All the male breast tumours were histologically verified and reclassified. The most common morphological type was infiltrating ductal carcinoma NOS, with other types constituting less than 20% of the cases. More than 80% of the infiltrating ductal carcinomas were of grades II and III. The mean age at diagnosis for the males was 66.2 years (range 42–87 years). DNA extracted from blood was available for 14 of the cases, but for all other cases DNA was extracted from archival tissue. DNA was extracted from blood samples using a standard phenol/chloroform method and from archival tissue using methods previously described (Wright and Manos, 1990).

For comparison were 309 male controls. Their mean age at the time of sample collection (1989–1995) was 52 years (range 18–88 years) and all were disease free by the end of the year 2000 according to the Icelandic Cancer Registry. DNA was extracted from blood, either using a standard phenol/choroform method or from frozen lymphocytes using a direct proteinase K digestion.

The female cases and controls have been previously described (Gudmundsdottir *et al*, 2001). Briefly, a total of 500 Icelandic females constituted the female breast cancer group, unselected with respect to family history. They were diagnosed with breast cancer in the years 1989–1995, with a mean age at diagnosis of 58.5 years (range 28–94 years). Samples were collected at the time of diagnosis. DNA was extracted from 455 blood samples and 45 tumour samples using a standard phenol/chloroform method.

A total of 23 female breast cancer patients, known to be BRCA2 mutation carriers, were added to the study to increase statistical power. These carriers come from another ongoing study that includes all women in Iceland who have been diagnosed with breast cancer.

The control group consisted of 395 females. Their mean age at the time of sample collection (1989–1995) was 49 years (range 25–82 years) and they were disease free by the end of the year 2000 according to the Icelandic Cancer Registry. DNA was extracted from blood, either using a standard phenol/choroform method or from frozen lymphocytes using a direct proteinase K digestion.

The study population, both cases and controls, is of a homogenous ethnical background. All blood samples come from the Icelandic Cancer Society's Biological Specimen Bank. Tumour samples were obtained from the Department of Pathology, National University Hospital of Iceland. All samples were precoded and stripped of personal identifiers in accordance with the standards of the Icelandic Data Protection Authority.

### Laboratory analysis

For the CYP17 genotyping, a 459 bp fragment was amplified using primers as previously described (Feigelson *et al*, 1997). PCR reactions were carried out in 15 *μ*l aliquots containing 25 ng of DNA, 10 pmol of each primer, 0.2 mM of each dNTP, 1.5 mM MgCl_2_ and 0.36 U Dynazyme polymerase (Finnzymes Oy, P.O. Box 148, FIN-02201, ESP00, Finland). Amplification conditions were 94°C for 3 min, followed by 30 cycles of 94°C for 30 s, 57°C for 30 s, 72°C for 1 min and a final step at 72°C for 5 min. The PCR products were digested with 2 U of MspA1 (NEB) for 3 h at 37°C in a final volume of 25 *μ*l and separated on 3% agarose gel (NuSieve 3 : 1) or on 7.5% nondenaturant acrylamide gel. A 150 bp fragment was amplified from DNA from archicval samples using primers as previously described (Young *et al*, 1999). PCR reactions were carried out as described above. Amplification conditions were 94°C for 3 min, followed by 40 cycles of 94°C for 45 s, 60°C for 45 s, 72°C for 1 min and a final step at 72°C for 5 min. PCR products were digested as described above and separated on 7.5% non-denaturant acrylamide gel.

BRCA2 mutation analysis was carried out by exon 9 amplification using the forward primer 5′-AAAGTCTGAAGAAAAATGATAGATTTA-3′ and the reverse primer 5′-AAAACCTGTAGTTC-AACTAAACAG-3′ (Tavtigian *et al*, 1996; Sigurdsson *et al*, 1997). The 999del5 mutation carriers were identified by an additional band, 5 bp smaller than the normal fragment, on 6% denaturant acrylamide gel or by the formation of heteroduplexes between the two fragments on a 7.5% non-denaturant acrylamide gel.

### Statistical analysis

Genotype proportions were compared between groups using the *χ*^2^ test, with Yates' correction, or the Fishers' exact test when appropriate. Tests for the Hardy–Weinberg equilibrium were conducted by comparing observed and expected genotype frequencies using a *χ*^2^ test. All analyses were performed using the statistical software package STATA 6.0 (STATA Corporation, College Station, TX, USA).

## RESULTS

The distribution of the CYP17 genotypes was compared between the male breast cancer cases and 309 male controls (Table 1

**Table 1:** Associations between CYP17 genotype and male breast cancer according to BRCA2 status

	**TT**	**TC**	**CC**
*All*			
Cases, *n* (%)	12 (30.8)	18 (46.1)	9 (23.1)
Controls, *n* (%)	103 (33.3)	160 (51.8)	46 (14.9)
OR (95% Cl)	1.0	0.97 (0.45–2.09)	1.68 (0.66–4.26)
*BRCA2 status of cases*			
999del5, *n* (%)	4 (26.7)	6 (40.0)	5 (33.3)
wt, *n* (%)	8 (33.3)	12 (50.0)	4 (16.7)
OR (95% Cl)	1.0	1.00 (0.21–4.71)	2.50 (0.42–14.84)

). No significant difference in genotype frequencies was observed between the two groups, although the frequency of the CC genotype was somewhat higher among cases (23.1%) than controls (14.9%) (*P*=0.32). No significant departures from the Hardy–Weinberg equilibrium were observed for the CYP17 genotypes among controls (*P*=0.66) or cases (*P*=0.90).

In all, 15 of the male cases were positive for the 999del5 mutation in the BRCA2 gene or 38.5%. The male breast cancer cases were divided into two groups according to their BRCA2 status and CYP17 genotype frequencies compared between them (Table 1). The CC genotype was more frequent among the carriers (33.3%) as compared to the noncarriers (16.7%), although this was not statistically significant (*P*=0.40).

Possible association of the CYP17 polymorphism with female breast cancer was also investigated by comparing the genotype frequencies between 500 breast cancer cases and 395 female controls (Table 2

**Table 2:** Associations between CYP17 genotype and female breast cancer according to BRCA2 status

	**TT**	**TC**	**CC**
*All*			
Cases, *n* (%)	193 (38.6)	247 (49.4)	60 (12.0)
Controls, *n* (%)	156 (39.5)	173 (43.8)	66 (16.7)
OR (95% Cl)	1.0	1.15 (0.86–1.54)	0.73 (0.49–1.11)
*BRCA2 status of cases*			
999del5, *n* (%)	20 (36.4)	28 (50.9)	7 (12.7)
wt, *n* (%)	133 (40.2)	162 (48.9)	36 (10.9)
OR (95% Cl)	1.0	1.15 (0.62–2.13)	1.29(0.51–3.30)

). No significant differences in genotype frequencies were observed between the two groups. The results did not change when the case group was divided into cases diagnosed before the age of 45 or after the age of 55 (results not shown). No statistically significant departures from Hardy–Weinberg equilibrium were observed for the CYP17 genotypes among controls (*P*=0.61) or cases (*P*=0.63).

Of the 500 cases, 363 were analysed for BRCA2 status. Of them, 32 (8.8%) were carriers of the 999del5 mutation. To increase the power of the analysis, 23 BRCA2 carriers diagnosed with breast cancer were added to the group. The CYP17 genotype frequencies were compared between the 55 carriers and 331 noncarriers, but no difference was observed between the two groups (Table 2).

Information about the stage of the disease was available for 481 patients out of 500 (Table 3

**Table 3:** Associations between CYP17 genotype and female breast cancer according to the stage of the disease

	**TT**	**TC**	**CC**
Controls, *n* (%)	156 (39.5)	173 (43.8)	66 (16.7)
*Cases by stage*			
Stage I	65 (35.7)	96 (52.7)	21 (11.5)
OR (95% Cl)	1.0	1.33 (0.91–1.95)	0.76 (0.43–1.35)
Stage II	98 (40.0)	117 (47.8)	30 (12.2)
OR (95% Cl)	1.0	1.08 (0.76–1.52)	0.72 (0.44–1.19)
Stage III+IV	15 (30.6)	27 (55.1)	7 (14.3)
OR (95% Cl)	1.0	16.2 (0.83–3.16)	1.10 (0.43–2.83)

). Five of those were classified as stage 0 and were not included in this part of the study. No association was observed between the CYP17 polymorphism and the stage of the disease among female breast cancer patients.

## DISCUSSION

To our knowledge, this is the first study to look for possible modifying effects on breast cancer risk among male BRCA2 mutation carriers. Young *et al* (1999) have reported a significant association of the C allele with increased risk of male breast cancer in a small study of 64 cases and 84 controls. Inevitably, the cases in this study are also few, but to increase the power of the study we used a larger control group of 309 individuals, giving 90% power to detect an OR⩾1.5 for carriers of at least one C allele, which is enough power to detect the increased risk of 2.1 reported by Young *et al* (1999). Despite that, our results do not indicate an association between the CYP17 polymorphism and risk of male breast cancer. We observed a slight increase in frequency of the CC genotype among the cases (23.1%) compared to controls (14.9%), but the difference did not reach statistical significance. It is quite interesting to note that the observed increase in frequency seemed mainly to be attributed to BRCA2 mutation status, as the CC genotype frequency was 33.3% among carriers but 16.7% among noncarriers, but again the difference was not statistically significant.

In females, we did not find any significant association between the CYP17 polymorphism and breast cancer risk, irrespective of menopausal status or stage of the disease, which is in agreement with most studies published so far (Dunning *et al*, 1998; Helzlsouer *et al*, 1998; Weston *et al*, 1998; Haiman *et al*, 1999; Nedelcheva Kristensen *et al*, 1999). In the first study published on this matter, Feigelson *et al* (1997) reported an increased risk of advanced breast cancer in women carrying a C allele (OR=2.5, 1.07–5.94). We were unable to confirm those results, despite having 90% power to detect OR⩾1.5 for carriers of at least one C allele when comparing genotype frequencies between controls and cases with stage III+IV of the disease. To our knowledge, the only other paper showing an association with the stage of the disease is that of Mitrunen *et al* (2000), who found a decreased risk of advanced breast cancer in association with the C allele among premenopausal women (OR=0.58, 0.31–1.07). Our results suggest that the T–C polymorphism in the CYP17 gene does not contribute to breast cancer risk in females overall or to the stage of the disease.

We were also interested to see whether the CYP17 polymorphism could act as a modifier of breast cancer risk in female BRCA2 mutation carriers. According to our results this does not seem to be the case, which is in agreement with the results of Spurdle *et al* (2000).

It is of special interest to examine possible modifying effects of different polymorphism on cancer risk in BRCA2 carriers in the Icelandic population. The reason is that only one BRCA2 mutation has been found and it shows very varied penetrance and expression (Thorlacius *et al*, 1997). It could be that an increase in oestrogen levels, possibly associated with the C allele of the CYP17 gene, could increase the risk of breast cancer in BRCA2 mutation carriers. BRCA2 mutated cells are not as efficient in repairing DNA damage as normal cells (Patel *et al*, 1998) and thus increased oestrogen levels could augment the mutation frequency even further. It would be interesting to investigate whether polymorphisms in other oestrogen metabolism genes would influence breast cancer risk in BRCA2 carriers.

In conclusion, our results do not suggest a major role for the CYP17 promoter polymorphism in breast cancer risk in general among males or females, but we were not able to exclude an association with breast cancer risk in male BRCA2 carriers because of the small sample size.
